# Below the Surface: IGF-1R Therapeutic Targeting and Its Endocytic Journey

**DOI:** 10.3390/cells8101223

**Published:** 2019-10-09

**Authors:** Caitrin Crudden, Dawei Song, Sonia Cismas, Eric Trocmé, Sylvya Pasca, George A. Calin, Ada Girnita, Leonard Girnita

**Affiliations:** 1 Department of Oncology-Pathology, Cellular and Molecular Tumor Pathology, Karolinska Institute, and Karolinska University Hospital, 17164 Stockholm, Sweden; c.crudden@amsterdamumc.nl (C.C.); Dawei.Song@ki.se (D.S.); oanasonia.cismas@ki.se (S.C.); eric.trocme@sll.se (E.T.); sylvya.pasca@stud.ki.se (S.P.); ada.girnita@ki.se (A.G.); 2 Department of Pathology, Cancer Centre Amsterdam, Amsterdam UMC, VU University Medical Centre, 1081 HZ Amsterdam, The Netherlands; 3 St. Erik Eye Hospital, 11282 Stockholm, Sweden; 4 Department of Experimental Therapeutics, The University of Texas, MD Anderson Cancer Center, Houston, TX 77030, USA; gcalin@mdanderson.org; 5 Center for RNA Interference and Non-Coding RNAs, The University of Texas MD Anderson Cancer Center, Houston, TX 77030, USA; 6 Dermatology Department, Karolinska University Hospital, 17176 Stockholm, Sweden

**Keywords:** insulin-like growth factor type 1 receptor, insulin receptor, RTK, GPCR, internalization, endocytosis, endosome, biased signaling, ubiquitination, β-arrestin

## Abstract

Ligand-activated plasma membrane receptors follow pathways of endocytosis through the endosomal sorting apparatus. Receptors cluster in clathrin-coated pits that bud inwards and enter the cell as clathrin-coated vesicles. These vesicles travel through the acidic endosome whereby receptors and ligands are sorted to be either recycled or degraded. The traditional paradigm postulated that the endocytosis role lay in signal termination through the removal of the receptor from the cell surface. It is now becoming clear that the internalization process governs more than receptor signal cessation and instead reigns over the entire spatial and temporal wiring of receptor signaling. Governing the localization, the post-translational modifications, and the scaffolding of receptors and downstream signal components established the endosomal platform as the master regulator of receptor function. Confinement of components within or between distinct organelles means that the endosome instructs the cell on how to interpret and translate the signal emanating from any given receptor complex into biological effects. This review explores this emerging paradigm with respect to the cancer-relevant insulin-like growth factor type 1 receptor (IGF-1R) and discusses how this perspective could inform future targeting strategies.

## 1. Introduction

Inter-cell communication is at the very heart of organism function. As our single-celled ancestors evolved to more and more complex organisms, the development of more specialized, specific, and fine-tuned cellular signaling tools was essential. Many of these long-evolved core systems that control cell survival/growth homeostasis are seized upon in the initiation and the maintenance of the malignant phenotype [1]. Among them, the receptor for the insulin-like growth factor 1 (IGF-1R) is one of the most evolved tools. The IGF-1R, the insulin receptor (IR), the insulin-related receptor (IRR) [2], and the most recently added IR/IGF-1R hybrid receptor [3,4] share a common ancestor that can be traced back 600 million years [5,6,7]. This primordial receptor was activated by only one ligand yet orchestrated multiple cellular functions from cell growth and metabolism to proliferation and survival. Somewhere along the evolutionary timeline towards mammals, both the primordial receptor and its singular ligand sustained a series of duplications that led to the development of several specialized receptors and ligands within the insulin/insulin-like growth factor (IIGF) family [8,9]. In mammals, the IR preferentially binds insulin and is involved in the regulation of cellular metabolism. The IGF-1R preferentially binds IGF-1 and IGF-2, although it can also be activated by insulin, and is implicated mostly in cellular proliferation, differentiation, and survival.

Considering the IGF-1R’s key roles in fundamental biological processes, it is not surprising that it is frequently found to be hijacked by awry oncogenic processes [10,11,12]. Extensive experimental and epidemiological studies have thoroughly documented the link between malignancy and IGF-1R across the majority of human cancer types. Not only is IGF-1R expression necessary for malignant transformation by numerous major oncogenes, but receptor inhibition (either by suppressing expression or activation) has been shown to lead to tumor cell growth inhibition [13,14,15]. As such, the IGF system has emerged as an obvious target for cancer therapy, fueling development of several anti-IGF-1R drugs and subsequent clinical trials [16,17,18,19]. In light of the structure-centered classification of the IGF-1R as a receptor tyrosine kinase (RTK) (for extensive review, see [11]) early in the drug development phase, inhibition of its kinase signaling was deemed the best anti-cancer strategy. To achieve this goal, several small-molecule kinase inhibitors and agents preventing ligand–receptor interaction were developed (Table 1) [20,21]. Most of the kinase inhibitors were soon abandoned, as they interfered with cell metabolism by also inhibiting the IR. Within the category of agents preventing ligand–receptor interaction, quite a few anti-IGF-1R antibodies were developed, and their efficacy tested in cell systems by using receptor phosphorylation as the main read-out. The most effective in preventing IGF-1R auto-phosphorylation were selected to be tested in clinical settings, yet in spite of promising preclinical data, clinical trials did not deliver the expected results [19,22,23]. This failure has led the field to question: is the inconsistency between outcomes in clinical and experimental settings a result of drug ineffectiveness or ultimately the wrong target? [10,17,19,22].

It was proposed more than a decade ago that IGF-1R downregulation (i.e., removal of the receptor from the cell surface) was a pre-requisite for any knock-on anti-neoplastic effects [13,24,25] and hence, the concept of receptor downregulation began to look more and more like the missing piece of the IGF-1R targeting puzzle. In this review, we examine IGF-1R downregulation and endocytosis as related to the rise and fall of IGF-1R targeted cancer therapy [22].

## 2. Receptor Internalization: The RTK Route

RTK endocytosis, i.e., the internalization and the trafficking of receptors inside the cell, was long believed to solely serve to terminate RTK signaling. However, more recent studies have demonstrated that RTKs actually continue to signal along the endocytic pathway. Hence, it is acknowledged today that the endocytic platform acts more as a master-regulatory device governing the spatio-temporal signaling, the distribution, and ultimately the biological outcome.

Plasma membrane receptors start their life-span synthesized in the endoplasmic reticulum and are then transported through the Golgi apparatus and delivered to the plasma membrane. Here, mature receptors accumulate, primed for their function to sense the extra-cellular environment. Eventually, plasma membrane receptors undergo endocytosis (internalization), by which they re-enter the cell, pass through the acidic endosomal system, and are processed for either recycling (returned to the plasma membrane) or undergo lysosomal or proteosomal degradation. Some degree of basal turnover is likely to go on with all receptors, which is thought to maintain receptor homeostasis at the plasma membrane. Some receptors internalize independently of a ligand (e.g., the transferrin receptor) [60,61,62]; however, receptor internalization is primarily ligand-dependent. Constitutive (ligand-independent) receptor endocytosis is a slow process and occurs at a similar rate to other membrane proteins [63]. Altogether, the rates of constitutive internalization, recycling, and degradation determine the half-life of an RTK and can vary greatly depending on receptor, cell type, and patho-physiological condition. For example, the half-life of the colony stimulating factor 1 receptor (CSF-1R) in macrophages is less than 1 h [64,65], whereas for the epidermal growth factor receptor (EGFR) in carcinoma cells, it is 24 h [66]. The IIGF family receptors are likely somewhere in the middle, with estimates around 6–7 h [67]. Canonical internalization of RTKs occurs after the binding of their respective ligands. Ligand binding causes auto-phosphorylation of the RTK intracellular tyrosine kinase domain as well as activation of the internalization signals (e.g., the conjugation of ubiquitin), allowing recruitment of adaptor molecules for the endocytosis machinery. For this reason, RTK internalization is often recognized as being coupled to kinase activation; ligand binding initiates both processes, greatly intensifying internalization rates, and is ultimately responsible for the downregulation of RTK populations [68,69,70].

### 2.1. Receptor Ubiquitination

Originally believed to serve as a label for protein degradation, it is now well accepted that the ubiquitin tagging of receptors serves as a sorting signal that directs subcellular trafficking. Ubiquitination is the covalent attachment of a 7 kDa ubiquitin polypeptide to lysine residues of a target protein. This process is orchestrated by the sequential action of E1, E2, and E3 ligase enzymes [71]. E1 and E2 load E3 with the ubiquitin, and E3 transfers it to the target protein and hence provides substrate specificity [72]. Proteins can be mono-ubiquitinated (addition of a single ubiquitin moiety), multi-ubiquitinated (at multiple lysine residues), or poly-ubiquitinated (addition of ubiquitin chains) [73,74]. Poly-ubiquitination can occur in a straight chain or branched, depending on which of the lysine residues within one ubiquitin molecule the subsequent ubiquitin molecule is attached to. Old or damaged cytosolic proteins are labeled with a poly-ubiquitin chain, which is then recognized and degraded by the proteasome constructed of multi-subunit proteolytic enzymes situated in the cytoplasm. In addition to the degradation of cytosolic proteins, ubiquitination tagging serves as a barcode to dictate the trafficking and the ultimate fate of the RTK through endosomal sorting. All major RTK subtypes are ubiquitinated upon growth factor stimulation. They can be mono-ubiquitinated or poly-ubiquitinated, and the type of modification regulates their ensuing fate [11,74].

Ubiquitin conjugation sites have been mapped within the IGF-1R, and mutational analysis studies have characterized their roles in subcellular trafficking [11,69,75,76]. Sepp-Lorenzino et al. [77] described IGF-1R degradation by a proteasome-mediated route in order to explain Herbymicin A-induced IGF-1R downregulation. Herbymicin A promoted IGF-1R degradation only in the presence of a functional ubiquitin E1 enzyme. Further, its action was prevented by proteasome inhibitors but insensitive to lysosomal inhibitors. Since then, four distinct E3 ubiquitin ligases have been recognized to promote IGF-1R ubiquitination and its subsequent degradation: Mdm2 [76], Nedd4 [78], c-Cbl [79], and HRD1 [80]. This complexity suggests a function-dependency to the ubiquitination processing [68]. The majority of reports thus far indicate that ubiquitination of the IGF-1R is mostly a ligand-dependent process—stimulation with IGF-1 rapidly induces receptor ubiquitination (within 5 min) with subsequent endocytosis. This can be seen on a receptor population level after about 6 h as detectably lower IGF-1R expression levels in whole cell lysates [69,79]. To unpick the interplay between the E3 ubiquitin ligases, Sehat et al. reported that low doses of IGF-1 (5 ng/mL) led to Mdm2-mediated ubiquitination, whereas high doses (50–100 ng/mL) led to c-Cbl-mediated receptor ubiquitination [79]. Mechanistically, Mdm2 and c-Cbl are RING-type E3 ligases, making them capable of acting alone, whereas Nedd4 is a HECT E3 ubiquitin ligase. However, it has been demonstrated that Mdm2 and Nedd4 bind the IGF-1R via the adaptor proteins β-arrestin [81,82] and Grb 10 [83], respectively. Such additional control layers suggest that the adaptor proteins may determine substrate specificity [68].

The placement and the arrangement of ubiquitin molecules encodes instructions for further receptor processing. Mdm2 has been shown to attach K63-conjugated ubiquitin chains, whereas c-Cbl attached K48-conjugated ubiquitin chains [79]. This divergence can then be followed in the subsequent internalization routes and receptor fate [68].

### 2.2. Clathrin-Dependent and Independent Receptor Internalization

Ubiquitinated receptors are brought back into the cell via the formation of pits, which burrow inwards from the plasma membrane. Upon ligand activation, many RTKs are found to localize to clathrin-coated pits [84,85,86]. Internalization can be blocked by chemical inhibitors of clathrin and small interfering RNAs (siRNA) against clathrin heavy chain. Such studies conclude clathrin-mediated endocytosis to be the predominant internalization route for this receptor family. Proteins that contain ubiquitin-interacting motifs (UIMs), such as epsin and Eps15, scaffold the receptor to components of the clathrin coat, e.g., AP-2 (appendage domain) as well as the terminal domain of clathrin heavy chain. In this way, clathrin coat components entrap their activated-RTK pit cargo. In the most well studied example, the EGFR–ligand complex can be detected in clathrin-coated vesicles 2–5 min after EGF stimulation [87,88,89,90]. Rate elucidation studies have demonstrated that clathrin-mediated endocytosis is the fastest pathway but can become saturated if a large number of receptors are activated. In such cases, a slower clathrin-independent endocytosis contributes significantly [91]. This is evidenced in that siRNA against clathrin considerably impairs EGFR internalization only when low EGF is used [92]. Similar domains and pathway compensations were then reported for both the IR and the IGF-1R [79,93,94]. Clathrin-independent pathways are of two main types; early studies described a macropinocytosis-like process involving actin cytoskeletal rearrangements and membrane ruffling [87]. It is worth noting that studies have shown IGF-1R modulation by elements of the adhesion-associated protein complexes, including the discoidin domain receptor 1 (DDR1) [95], a non-integrin collagen RTK, and the non-receptor tyrosine adhesion kinase FES-related (FER) [96], meaning that the extracellular matrix and the adhesion signaling also contribute to IGF-1R subcellular trafficking. The second route is defined through its sensitivity to inhibitors of caveolae and cholesterol-disrupting agents [79,92,97]. Confocal microscopy with immuno-fluorescent localization demonstrates that the IGF-1R can localize with both the lipid raft caveolar marker tyrosine phospho-caveolin-1 (pY14) and also the early endosome marker EEA-1, meaning it can be internalized via both clathrin-dependent or clathrin-independent (caveolar) routes [79]. Aligning with context-dependent ligase recruitment, co-localization of the IGF-1R with phospho-caveolin occurs at high IGF-1 doses (100 ng/mL) and is enhanced by c-Cbl overexpression and decreased by Mdm2 overexpression. On the other hand, IGF-1R/clathrin co-localization occurs at low IGF-1 (5 ng/mL) doses and is enhanced by Mdm2 overexpression and inhibited by c-Cbl overexpression [79]. One possible scenario is sequential ligase action, with Mdm2 predominance at physiological ligand concentrations followed by c-Cbl at higher ligand concentrations that saturate the clathrin-dependent internalization route [69,79]. This plethora of E3 ligases and receptor processing ultimately ensures flexibility to IGF-1R function for specific cellular needs.

After internalization of the receptor–ligand complex, the next steps involve the turnover of components. Using selective inhibitors and mutational studies, the sorting mechanisms begin to be pieced together. Members of all RTK subfamilies undergo this agonist-triggered accelerated lysosomal/proteasomal degradation, and therefore ligand-dependent global receptor downregulation is a hallmark of this receptor family. Overall, a lysosomal inhibitor has a much greater impact on wild-type IGF-1R degradation than a proteasome inhibitor, indicating that the IGF-1R is predominantly degraded through the lysosome and less by proteosomal action. It is, of course, feasible that part of the receptor is degraded via the proteasome and part is degraded lysosomaly. ATP-deficient mutants are not degraded at all, supporting the model of ligand-dependency and/or phosphorylation requirement [10,69,79]. In addition, the C-terminal tail of the receptor is a requirement for ubiquitination—a receptor that is functional in kinase activity but harbors a c-terminal tail truncation cannot be ubiquitinated [10,69,79,81,83,98,99]. Increasing the complexity of the system, ubiquitin-mediated control goes beyond the receptor to its docking station (e.g., IRS) and downstream signaling effectors [100]. The layers of regulation imposed upon the IGF-1R’s intracellular journey by ubiquitination were recently extensively reviewed in [68].

### 2.3. Subcellular Receptor Trafficking

Clathrin-dependent and -independent endocytosis both deliver receptor–ligand cargo to early endosomes located in the cell periphery. In most cases, the receptor–ligand complex remains intact, although the ligand can dissociate in the acidic environment of the endosome. In such cases, the released ligands are contained in the vesicular part of the endosome, whereas unoccupied receptors congregate in tubular extensions (membrane area) [63].

From this point, receptors can rapidly recycle back from early endosomes to the plasma membrane in a process known as back fusion. As the early endosome matures and moves towards the peri-centriolar region, its biochemical composition changes with increasing luminal acidification. The membrane invaginates inwards to create intra-luminal vesicles (ILVs), at which point the endosome is referred to as a multi-vesicular body (MVB) [101]. At this level, membrane invaginations have shifted the RTKs into the intra-luminal vesicles. If destined for recycling, RTKs can also be delivered to Rab11-containing recycling compartments; however, this is generally a slower route (30–60 min) than back fusion from earlier endosomes (2–5 min) [102]. As endosomal maturation continues, recycling cargo and early markers such as Rab5 and EEA1 are lost, and late endosomal markers such as Rab7 enrich [103]. Late endosomes fuse with primary lysosomes, and RTKs that reach this point are degraded by proteolytic enzymes. It is worth noting that many studies in this field do not dissociate recycling from degradation, and therefore reported rate alterations are likely composite images of changes in the relationship between the two intertwined. The specific contribution of receptor recycling on expression levels is rarely considered for the RTK family. However, one study does attribute considerable recycling to the IR system [104].

Another fate of the endocytosed RTK is the fusion of MVBs with the plasma membrane and the release of its contents as “exosomes”. Discovered in the context of removal of the transferrin receptor [105] during maturation of reticulocytes and termed “selective externalization”, this process has now been demonstrated for many physiological and pathological instances/cargo. Many RTKs have been reported to be released by cells in this way, including EGFR [106,107,108,109], IGF-1R [110,111], and MET [112]. The intricacies of how this mediates inter-cellular signaling and expands the signal profile of RTKs are just starting to be understood. Research has intensified in this area due to the discovery that cancer cells upregulate exosome production, and their cargo can have cancer-promoting effects on recipient cells. For example, exosomes released from melanoma cells that carry MET educate bone marrow progenitor cells towards a pro-metastatic phenotype [112].

RTKs and their endosomal sorting machinery were heavily investigated in close association with their kinase activity and due to their involvement in oncogenesis. Yet, over the last two decades, experimental, clinical, and epidemiological data clearly demonstrate that RTKs operate in a close relationship with the larger super-family of G protein-coupled receptors (GPCRs) in a wide range of physiological and pathological processes. The GPCR family has its own trafficking process orchestrated by the β-arrestin/G protein-coupled receptor kinase (GRK) system. The more knowledge we garner in this context demonstrates that the cross-talk between the two receptor systems goes beyond transactivation to the very heart of GPCRs’ internalization machinery [10,23,113,114,115].

## 3. Receptor Internalization: The GPCR Route

GPCRs, also known as 7 transmembrane domain receptors, comprise the largest family of cell surface receptors in mammalian cells. They are functionally much more diverse than the RTKs, spanning nearly every physiological process in the human body from nerve transmission to hormone signaling [116,117]. As evidence to their critical roles in controlling physiological and pathological processes, at least a third of the drugs approved within the last decades target GPCRs [116,118,119,120]. Yet, in spite of their dominance in the drug discovery field, a rather small fraction have anti-neoplastic indications—only 4.4% of the drugs on the market (21/475 in 2017) and 7.1% of the agents presently investigated in clinical trials (23/321 in 2017) [116]. They receive relatively minor attention when compared to the small-scale family of RTKs, of which targeting agents with cancer indications are approved every year. For instance, in 2015, there were 21 approved drugs only in the category of small molecule tyrosine kinase inhibitors, while many more were evaluated in advanced-phase clinical trials [121]. The number of studies assigning GPCR roles in oncogenesis is growing rapidly in recent years; therefore, it may be reasonable to claim that GPCRs’ current position in anti-cancer drug development is just the tip of the iceberg and that their therapeutic potential is narrowly exploited.

**Figure 1:**
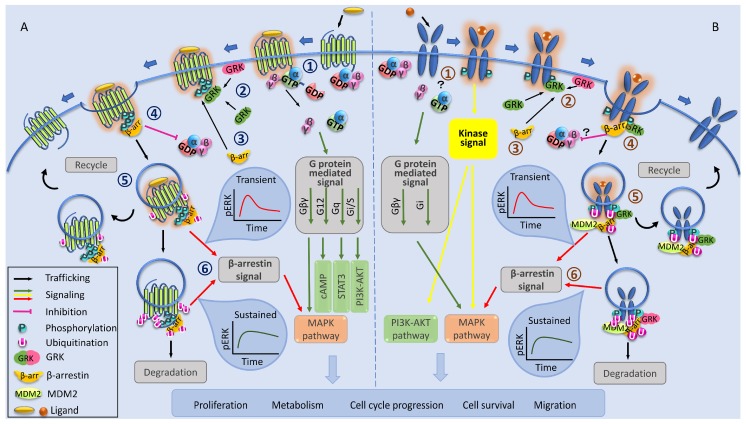
IGF-1R as a receptor tyrosine kinase (RTK)/G protein-coupled receptors (GPCR) functional hybrid model. (**A**) Classical mechanism of signaling activation and receptor trafficking of GPCRs: (1) Ligand-binding induced receptor activation leads to GDP exchange for GTP on the G protein α subunit, resulting in the dissociation into Gα and Gβγ subunits. G protein subunits then interact with second effector proteins to promote several downstream pathways. (2) The increased concentration of Gβγ subunits initiates G protein-coupled receptor kinase (GRK) recruitment. (3) GRKs phosphorylate the receptor at c-terminal serine/threonine residues, recruiting β-arrestins. (4) β-arrestins bind to the phosphorylated receptor, preventing G protein coupling and impeding further G protein signaling. (5) The receptor becomes desensitized and is internalized and trafficked through recycling or degradation pathways. (6) By acting as a scaffold, β-arrestin initiates a second wave of downstream signaling [mitogen-activated protein kinase (MAPK) is illustrated]. (**B**) IGF-1R shares GPCR functionality in signaling and trafficking: (1) The ligand-activated IGF-1R facilitates G protein subunit dissociation and subsequent downstream G protein signaling. In parallel, receptor autophosphorylation activates classical kinase signaling. (2) GRKs phosphorylate the receptor at C terminal serine residues. (3) β-arrestins are recruited, which (4) prevents further G protein coupling and initiates the desensitization and the internalization of the receptor. (5) Once internalized, the receptor is directed for recycling or degradation. (6) β-arrestins control the activation of secondary wave of kinase-independent signaling.

Despite the staggering diversity of extracellular signals that they respond to, mechanistically, GPCRs share remarkably similar machinery for signaling activation and receptor trafficking [11,113,114,122,123]. The classical paradigm (Figure 1A) describes GPCRs carrying out a six-phase functional journey, including G protein signaling activation and GRK-dependent phosphorylation of serine residues of the receptor, which triggers β-arrestin recruitment with subsequent receptor desensitization, internalization/trafficking, and a second signaling wave [11,81,124,125,126,127].

### 3.1. G Protein Signaling

Unlike RTKs, GPCRs lack intrinsic catalytic activity and therefore rely on the interaction with their namesake G proteins for signaling activation. Ligand-binding to the GPCR induces conformational change within the receptor that promotes the coupling of heterotrimeric G proteins. The active receptor catalyzes the exchange of GDP to GTP on the G protein α subunit (Figure 1A) [128]. The heterotrimeric G proteins use this additional energy to dissociate into Gα and Gβγ subunits [129], thus triggering the classical “G protein signaling pathway”. Both effectors interact with downstream proteins to initiate several signaling branches, including the mitogen-activated protein kinase (MAPK) cascade, cyclic adenosine monophosphate (cAMP), Phosphoinositide 3-kinase (PI3K)-Akt, and protein kinase A (PKA) (Figure 1A) [127,130]. This activity continues until the system initiates negative feedback to turn off signaling (G protein desensitization). One such feedback determinant, increased Gβγ concentration, triggers GRKs recruitment to the vicinity of the activated receptor, commencing the “turning off” process. G protein desensitization occurs because the GRK-mediated phosphorylation event promotes the enrollment of a family of proteins known as β-arrestins to the receptor, which physically interrupt the receptor-G protein coupling [123,131].

### 3.2. GRK/β-arrestin-Dependent Receptor Desensitization

The first GRK isoforms were discovered almost half a century ago, when proteins capable of desensitizing the rhodopsin photoreceptor and the β2-adrenergic receptor via phosphorylation were identified [132,133,134,135]. The human genome encodes seven GRK isoforms (GRK1–GRK7); GRK2/3/5/6 are ubiquitously expressed in all tissues, while GRK1/4/7 show specific expression [136]. All isoforms phosphorylate unique serine and threonine residues of the intracellular domains (third cytoplasmic loop) and/or the C-termini of activated receptors [131,137]. GRKs’ kinase domain active structure is stabilized by the docking interaction with the agonist-coupled GPCRs, and thus the phosphorylation of the substrates commences [138,139]. Based on their structural resemblance, the GRKs are grouped into three subfamilies [140]. The GRK1 family includes GRK1 and 7, prenylated at their C-termini to enable their membrane localization [139,141]. The GRK2 family includes GRK2 and 3, which display cytoplasmic localization and translocate to the membrane following association with heterotrimeric G protein βγ-subunits released upon receptor activation of G proteins [142,143]. Members of the GRK2 family share approximately 84% sequence similarity, containing a pleckstrin homology (PH) domain, which controls G protein mediated translocation [129,144,145]. The GRK4 family comprises isoforms 4, 5, and 6 positioned at the plasma membrane due to their ability for direct PIP2 binding [146]. Unlike other members of the GRK family, GRK5 and GRK6 can phosphorylate both active and inactive receptors [139,143]. All GRK-mediated phosphorylation engages the second major component of the system—a family of proteins named arrestins, as they were originally discovered to cease or “arrest” the G protein signaling [129,147].

There are four isoforms of arrestins (1–4) encoded in the human genome; arrestins 1 and 4 are solely expressed in the retinal tissue, while arrestins 2 and 3 (also known as β-arrestins 1 and 2, respectively) are ubiquitously expressed in all tissues [148]. Although β-arrestins 1 and 2 have a largely similar structure and can partially substitute for each other in knock-out mouse models [149,150], they can play similar, distinctive, or opposite roles in the regulation of GPCRs [151,152,153]. The GRK-dependent phosphorylation regulation of arrestin recruitment gave rise to the development of a “barcode hypothesis” [125,136]. By translating a specific receptor conformation into patterns of β-arrestin recruitment and interaction, GRKs are said to establish a barcode across serine and threonine residues on the C-terminal tail, thus regulating receptor functionality [154,155]. The two-step GPCR desensitization hypothesis whereby a family of Ser-Thr protein kinases (GRKs) specifically phosphorylate ligand-activated GPCRs, creating binding sites for arrestins to prevent further G protein recruitment, is termed heterologous desensitization (Figure 1A) [126,127,129,154,156,157,158,159,160]. 

### 3.3. Receptor Internalization, Trafficking, and Second-Wave Signaling

Once recruited to the transmembrane docking site, β-arrestins block the coupling sites for the G proteins, bringing their signaling to an end [123,131]. The desensitized receptor is internalized, which can lead to either recycling of the receptors to the plasma membrane or degradation. The wide repertoire of isoforms of the GRK/arrestin families allows for a spectrum of potential patterns (barcodes), which translate into distinct fates for the receptor complex [123,129,131,155].

β-arrestins promote GPCR endocytosis by mediating an interaction between the C-terminus and the heavy chain of clathrin and β-subunit of adaptor protein-2 (AP2) [139,161]. In much the same way as described earlier for RTKs, GPCRs are then concentrated in clathrin-coated pits, internalized, and finally follow steps in accordance with the isoform of β-arrestins employed in the downregulation. Although discovered and named for their signal arresting role, it is now clear that this under-represents reality. In addition to interrupting the receptor-G protein coupling and impairing the G protein signaling, β-arrestins 1 and 2 themselves activate their own signaling pathways, such as MAPK, PI3K, and NF-κB cascades, by acting as scaffold proteins [162,163,164,165,166,167]. Differential affinities for the β-arrestin isoforms separate GPCRs into two major classes. Class A members such as the dopamine D1A receptor, the μ-opioid receptor, and the β2 adrenergic receptor bind β-arrestin 2 with greater affinity than β-arrestin 1, recycle rapidly, and transiently activate MAPK. Class B members such as the angiotensin II type 1A receptor and the vasopressin V2 receptor bind both isoforms with equal affinity, recycle slowly, and sustain MAPK signaling [168,169,170,171].

Despite original models, it is now clear that internalization of a receptor does not necessarily mean immediate cessation of all associated signaling. Ligand-mediated endocytosis is multi-functional; although endosomal acidic dissociation of the ligand–receptor complex can attenuate any signal originating from it, the endosome can also facilitate the interaction between the internalized receptor and the downstream signaling molecules [172,173,174], best illustrated by the case of the arrestins. As multi-functional adaptor molecules, arrestins govern GPCR physiology, but does their affiliation lie solely with this receptor family?

## 4. IGF-1R as an RTK/GPCR Functional Hybrid

The work that identified Mdm2 as a novel E3 ubiquitin ligase for the IGF-1R [76,175] shed light on remarkable parallels between this receptor and the larger (but believed to be separate) family of GPCRs. The adaptor molecule that brings Mdm2 to the ligand-activated IGF-1R was discovered to be the master regulator of GPCR biology, β-arrestin [81,176]. This work showed that, similar to GPCRs, β-arrestins not only aid IGF-1R internalization but initiate their own second wave of signaling through the MAPK/ERK pathway (Figure 1B). Intriguingly, β-arrestin-mediated ERK activation occurs even in conditions with tyrosine kinase domain inhibited or mutated versions of the IGF-1R [49,81,177]. This dual role of β-arrestin 1 in the case of IGF-1R downregulation and signaling activation was reminiscent of its role in the GPCR family; while internalizing the ligand-activated receptor, β-arrestins also activate the MAPK pathway [11,68,81,178] (Figure 1B). Functional antagonism has also been revealed in regard to β-arrestin isoforms at the IGF-1R. Both isoforms co-immunoprecipitate with IGF-1R; however, the ligand-occupied receptor has greater affinity for β-arrestin 1. This association lasts longer and sustains MAPK/ERK signaling [81,98]. Conversely, β-arrestin 2 has greater affinity for the ligand-unoccupied receptor. This interaction is transient and can trigger receptor ubiquitination and degradation but without any signal activation [81,98] (Figure 1B).

**Figure 2:**
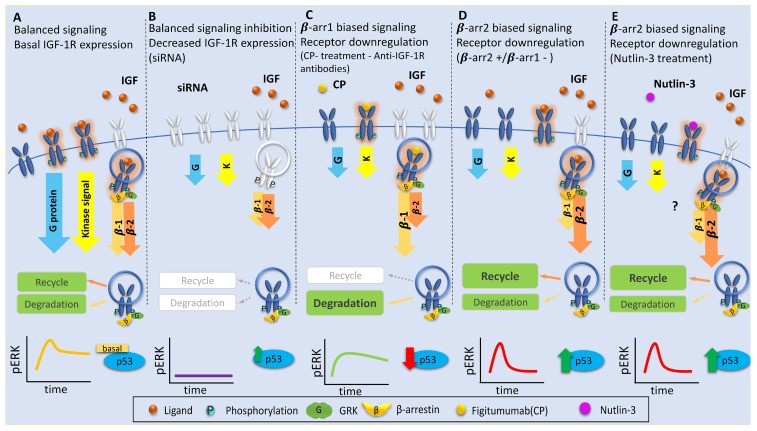
Therapeutic implications for IGF-1R downregulation as related to various types of associated signaling activation. (**A**) Balanced signaling: IGF-1 (balanced agonist) binds to the receptor and equally activates all downstream signaling in a balanced manner—G protein, kinase, and β-arrestin 1/β-arrestin 2 signaling (β-1/β-2). Receptors can then be either degraded or recycled. This results in moderate intensity pERK and basal p53 levels. (**B**) Balanced inhibition: small interfering RNAs (siRNA)/short hairpin RNAs (shRNA)-mediated IGF-1R depletion results in inhibition of all downstream signaling. This strategy diminishes pERK and slightly elevates p53 levels. (**C**) β-Arrestin 1 biased signaling: Anti-IGF-1R antibodies [Figitumumab, also known as CP-751871 (CP in the Figure)] treatment results in receptor degradation with β-arrestin 1-biased signaling and sustained pERK activity, and β-arrestin 1 signaling predominance maintains low p53 levels. (**D**) β-Arrestin 2 biased signaling (β-arrestin 2 overexpression/β-arrestin 1 inhibition): the receptor preferentially binds β-arrestin 2, generating a transient pERK signal and receptor recycling. β-arrestin 2 signaling (or absence of β-arrestin 1 signaling) increases p53 levels, possibly by sequestering both β-arrestin 1 and Mdm2 in the cytoplasm. (**E**) β-Arrestin2 biased signaling (Nutlin-3 treatment): small molecule Nutlin-3, a promising therapeutic option, activates transient ERK signaling and produces a boost of p53, mimicking the pattern observed upon β-arrestin 2 overexpression.

Recognized as a universal mechanism of GPCR regulation, β-arrestins bind to the receptor following phosphorylation of specific serine residues by the G protein-coupled receptor kinases (GRKs). This warranted investigation into the mechanism of β-arrestin binding to the IGF-1R, which revealed that GRK-mediated receptor phosphorylation coordinates this process [124] (Figure 1B). There seems to be contrasting roles between GRK2 and 6, whereby phosphorylation of serine residues on the receptor C-terminal tail by either isoform encodes a barcode for subcellular fate [124] (Figure 1B). Specifically, GRK2 phosphorylation promotes transient β-arrestin 2 binding and predominance for receptor recycling, whereas GRK6 promotes a stable receptor/β-arrestin 1 interaction that leads to receptor complex degradation [124] (Figure 1B). The body of work that uncovered this shared functionality also shed light on a new onco-relevant link between the IGF-1R and the crucial tumor suppressor, p53. Transcriptional links between the two pathways have been well established—wildtype but not mutant p53 suppresses IGF-1R gene transcription, part of the mechanism by which p53 can arrest the cell cycle [179,180,181]. The discovery of Mdm2/β-arrestin-mediated IGF-1R ubiquitination also exposed IGF-1R and p53 protein co-dependency, as they compete for the same E3 ligase, Mdm2 [76,81,175,176]. Furthermore, by controlling the relative expression of the arrestin isoforms, we demonstrated knock-on effects on p53 levels. Imbalance towards the β-arrestin 1 isoform via overexpression or silencing of β-arrestin 2 sustains MAPK signaling and keeps p53 at basal low levels (Figure 2C) [98,99]. The opposing scenario—imbalance towards β-arrestin 2—circumvents MAPK signaling and causes p53 levels to accumulate (Figure 2D), leading to cell cycle arrest and decreased viability of melanoma cells [98,99]. This scenario does, however, require functional p53, highlighting an interesting therapeutic scenario in the group of wildtype p53 cancers [98,99,182].

Initially suggested by the peculiar sensitivity of the IGF-1R to the Gi protein inhibitor, pertussis toxin [114,183], and having been fully explored by extensive studies since then, it is now clear that the IGF-1R makes direct use of all GPCR signaling components: G proteins, GRKs, and β-arrestins [11,39,68,81,98,124,176]. While examples of RTKs/GPGRs family crosstalk have been known for quite some time [15], this is distinct from that which can occur at the IGF-1R, whereby this receptor is directly utilizing GPCR components of G proteins, GRKs, and β-arrestins and can activate a signal cascade in a kinase domain-independent fashion. By all functional definitions, the IGF-1R has shown itself capable of classification as a functional GPCR. In respect of the evidence, we advocate that the IGF-1R should be regarded as an RTK/GPCR functional hybrid [10,11,23] and that this paradigm should be used for drug development. This is especially important, as targeting strategies designed under a kinase-only paradigm have already proven to be insufficient and thus outsmarted by this complex network.

## 5. Discussion: Therapeutic Implications

Over the last few decades, the potential of IGF-1R as a target for cancer treatment has been extensively investigated and almost exclusively aligned to the kinase-fits-all model. Nevertheless, the critical role of receptor removal from the cell surface was openly recognized from the beginning, which was exemplified when Renato Baserga stated, in 2005, “An antibody against the IGF-IR, to be effective, has to inhibit the binding of both IGF-1 and IGF-2, induce the downregulation of the receptor, and have little or no effect on the IR signaling” [13]. This concept, fundamentally true for all anti-IGF-1R strategies, emerged from the early antisense-based experimental work, which described complete inhibition of cancer cells growing in monolayer or as xenografts in animal models [13]. The corollary of these studies is that preventing the receptor’s de novo synthesis (e.g., antisense, siRNA) results in an overall decrease of IGF-1R expression and all of its signaling branches (Figure 2). This process is similar to the one employed by the cells in basal conditions in that it diminishes the downstream signaling in a *balanced* manner (Figure 2). In place of gene silencing (not yet possible in humans), kinase inhibitors or the antibodies-based strategy targeting IGF-1R are preferred in clinical settings. Just like anti-sense strategies, all antibodies and all kinase inhibitors against IGF-1R tested thus far in clinical trials (Table 1) were confirmed to preclude kinase-dependent signaling activation [verified as decreased phosphorylated-(p-)IGF-1R]. However, with the notable exception of picropodophyllin (PPP) [47,48,184,185], all kinase inhibitors had no effects on IGF-1R expression at the cell surface (Table 1). It is worth mentioning that, in the case of kinase inhibitors, both pERK and pAkt were employed to verify the inhibition of downstream signaling, and they were found to be decreased in a balanced manner (Table 1). Once more, PPP was the exception, demonstrating biased pERK activation linked to the downregulation process [49]. On the other hand, when it came to targeting antibodies, pAkt was always employed as a surrogate to verify decreased downstream IGF-1R signaling, whereas pERK was found to be reduced, increased, or was not investigated (Table 1). Follow up studies confirmed pAkt inhibition but discovered that pERK, in different experimental models, demonstrated a great degree of variability (Table 1).

Intriguingly, in contradiction with the classical paradigm postulating kinase activity/downregulation interdependency, all antibodies proved very effective at downregulating the IGF-1R (Table 1). This process occurred very fast in cell lines models (1–4 h) and was also confirmed in xenografts models (Table 1), yet the clinical results are far from what was expected. We and others demonstrated that antibody-induced IGF-1R downregulation stabilizes a *biased* receptor conformation that preferentially activates kinase-independent β-arrestin 1 signaling (Figure 2 and Table 1) and not only promotes MAPK enhancement but also represses the tumor suppressor p53 activation (Figure 2), which could explain the cancer cell survival, the augmented metastatic potential, and the overall limited response to this single agent therapy [10,39,98,99,182]. It should be noted here that there were some exceptions [186]. Firstly, most antibodies do show response in in vivo models, and secondly, clinical response to single-agent anti-IGF-1R is reported in some patients, particularly in Ewing’s sarcoma. A number of reasons are suggested for this unique efficacy, including that it derives from their genetic hallmark: the direct connection between their oncogenic fusion EWS/ETS transcripts and the IGF system [34,187,188,189]. In such cases, the aberrant EWS/ETS transcript likely influences IGF signaling to such a degree that the impact of an antibody shifts the balance differently than the norm. Whilst hoping that these few success cases could offer important insight into the mechanisms, anti-IGF-1R therapy is still yet to reach clinical practice in the treatment of Ewing’s sarcoma patients, nor any other cancer types [190]. The prerequisite for efficient targeting of receptor removal set against the reality that its downregulation triggers signaling sustaining the cancer-phenotype presents a problem with no apparent way out. However, a possible solution was revealed by studies demonstrating the molecular mechanism behind arrestin involvement, i.e., opposing behaviors of the β-arrestin isoforms on IGF-1R downregulation and signaling [98]. Both β-arrestins downregulate the receptor, however, β-arrestin 2 is more efficient in conditions with low ligand availability. Most importantly, such conditions promote a GPCR class A-like behavior of the IGF-1R with transient β-arrestin 2/receptor interaction and subsequent MAPK-biased signaling and eventually with p53 reactivation (Figure 2) [98,99,182]. While uncovering antagonism between the β-arrestin isoforms in controlling IGF-1R downregulation, it was demonstrated that biasing the IGF system toward β-arrestin 2 decreases the viability and the metastatic potential of cancer cells and hence could be considered an effective therapeutic strategy (Figure 2) [98,99,182]. As β-arrestin 2 is more efficient in downregulating the receptor in conditions with low ligand, another option could be to develop therapeutic strategies involving ligand sequestration (e.g., anti-IGF antibodies or IGF traps) whilst also tipping the arrestin balance toward β-arrestin 2.

It could be argued that destabilizing the β-arrestin 1/β-arrestin 2 equilibrium via transgenic approaches encounters the same limitations as transgenic downregulation of the IGF-1R. Then again, we have to consider the fact that Mdm2 co-orchestrates stress-induced survival pathways by acting as ubiquitin ligase for IGF-1R, p53 and β-arrestins. Within this scenario, we recently demonstrated that disruption of the Mdm2–p53 interaction by small molecule Nutlin-3 allows Mdm2 accumulation and triggers IGF-1R/Mdm2 association with subsequent receptor ubiquitination and downregulation [99]. Under such conditions, the MAPK signaling associated with receptor downregulation is biased towards βarrestin 2, is transient and not sufficient to provide protection for malignant cells (Figure 2) [98,99,182]. Add all of this to the reactivation of p53 and the possibility to control the β-arrestin recruitment via the GRK system and a comprehensive anti-tumorigenic cellular scenario arises.

## 6. Concluding Remarks

The central roles in cancer of RTKs in general and IGF-1R in particular have been known for many decades, and they serve as targets for many therapeutic approaches. In the post-trial years, various reasons have been suggested for the failure of first-line strategies, most highlighting the unappreciated complexity of the IGF-1R/IR system: including multiple ligands, hybrid receptors between the IGF-1R and the IR, nuclear translocation of components, cross-talk to other pathways, as well as a lack of patient selection markers, reasons which have been reviewed extensively elsewhere [17,18,22,190,191,192,193,194]. This review focuses on the IGF-1 and IGF-1R path due to its intense study in drug development pipelines, however, this is not intended to reflect the importance of IGF-2 or IGF-2R. IGF-2 is by far the most abundant peptide from the IIGF family in human circulation. Considering its much more limited study, it is reasonable to claim that we are vastly under-informed on the specific contribution of this arm to patho-physiology. This perspective is comprehensively reviewed by Holly et al. within this special focus issue [195].

All the while, the traditional kinase-only model of activation is still prevalent in drug development. With this review, we aim to draw a parallel with the more successful story of GPCR targeting, where the naive ON/OFF model was abandoned more than a decade ago. For RTKs in general and IGF-1R in particular, in addition to the classical kinase signaling, their ultimate biological effects are orchestrated by several other post-translational modifications, interactions, and biological processes. It is now clear that the endosome is deeply entrenched in growth factor receptor function. Defective vesicular trafficking of growth factor receptors, including an imbalance between recycling versus degradation and versus exosomal release, appears prevalent. Derailed endocytosis is thus emerging as a multi-factorial hallmark of cancer cells [196]. There is now a critical need for a widespread update to the working model that recognizes the intricacies of the IGF signaling system; only then will rational drug design, therapeutic combinations, and real clinical benefits match the decades of supportive experimental data in the field.

## Figures and Tables

**Table 1:** Effect of IGF-1R targeting on receptor signaling and downregulation.

Compound	IGF-1R Downstream Signaling					IGF-1R Downregulation	β-arr Signaling
	pIGF-1R	pAKT		pERK			
		Original Report	Follow up	Original Report	Follow up		
**IGF-1R Monoclonal Antibodies**							
Cixutumumab (IMC-A12)	↓ [20]	↓ [20]		↓ [20]	↓ [26]	Yes [20]	
Teprotumumab (R1507)	↓ [21]	↓ [21]		→ [21]	↑ [27]	Yes [21]	β-arr1 [27]
Dalotuzumab (MK-0646)	↓ [28]	NI	↓ [29,30]	NI	→ [30,31]	Yes [28,30]	
Ganitumab (AMG 479)	↓ [32]	↓ [32]		NI	→ [33]	Yes [32]	
Robatumumab (SCH717454)	↓ [34]	↓ [34]		↓ [34]	↓ [35]	Yes [34]	
AVE1642 (EM164)	↓ [36]	↓ [36]		↓ [36]	→ [37]	Yes [36]	
Figitumumab (CP-751)	↓ [38]	↓ [38]		NI	↑ [39] → [40]	Yes [38]	β-arr1 [39]
αIR3	↓ [41]	NI	↓ [42]	NI	↓ [43] ↑ [44]	NI	
**Tyrosine Kinase Inhibitors**							
BMS-536924	↓ [45]	↓ [45]		↓ [45]			
OSI-906 (linsitinib)	↓ [2]	↓ [2]		↓ [2]	↓ [46]	NI	
AXL1717 (PPP)	↓ [47]	↓ [47]		↓ [47]	↓ [48] ↑ [49]	Yes [50]	β-arr [49]
BMS-754807	↓ [51]	↓ [51]		↓ [51]	↓ [52]	NI	NI
AG-1024 (Tyrphostin)	↓ [53]	NI	↓ [54]	NI	↓ [55]	NI	NI
NVP-AEW541	↓ [56]	↓ [56]		↓ [56]	↓ [57,58]	NI	NI
KW-2450	↓ [59]	↓ [59]		↓ [59]		NI	NI

NI: Not investigated; ↓: inhibition; ↑: activation; →: no changes; β-arr: β-arr signal.

## References

[1] Zimmer C. (2007). Evolved for cancer?. Sci. Am..

[2] Ji Q.S., Mulvihill M.J., Rosenfeld-Franklin M., Cooke A., Feng L., Mak G., O’Connor M., Yao Y., Pirritt C., Buck E. (2007). A novel, potent, and selective insulin-like growth factor-I receptor kinase inhibitor blocks insulin-like growth factor-I receptor signaling in vitro and inhibits insulin-like growth factor-I receptor dependent tumor growth in vivo. Mol. Cancer Ther..

[3] Bailyes E.M., Nave B.T., Soos M.A., Orr S.R., Hayward A.C., Siddle K. (1997). Insulin receptor/IGF-I receptor hybrids are widely distributed in mammalian tissues: Quantification of individual receptor species by selective immunoprecipitation and immunoblotting. Biochem. J..

[4] Belfiore A., Frasca F., Pandini G., Sciacca L., Vigneri R. (2009). Insulin receptor isoforms and insulin receptor/insulin-like growth factor receptor hybrids in physiology and disease. Endocr. Rev..

[5] Wood A.W., Duan C., Bern H.A. (2005). Insulin-like growth factor signaling in fish. Int. Rev. Cytol..

[6] Skorokhod A., Gamulin V., Gundacker D., Kavsan V., Muller I.M., Muller W.E. (1999). Origin of insulin receptor-like tyrosine kinases in marine sponges. Biol. Bull..

[7] Ullrich A., Gray A., Tam A.W., Yang-Feng T., Tsubokawa M., Collins C., Henzel W., Le Bon T., Kathuria S., Chen E. (1986). Insulin-like growth factor I receptor primary structure: Comparison with insulin receptor suggests structural determinants that define functional specificity. EMBO J..

[8] LeRoith D., Kavsan V.M., Koval A.P., Roberts C.T. (1993). Phylogeny of the insulin-like growth factors (IGFs) and receptors: A molecular approach. Mol. Reprod. Dev..

[9] Hernandez-Sanchez C., Mansilla A., de Pablo F., Zardoya R. (2008). Evolution of the insulin receptor family and receptor isoform expression in vertebrates. Mol. Biol. Evol..

[10] Crudden C., Shibano T., Song D., Suleymanova N., Girnita A., Girnita L. (2018). Blurring Boundaries: Receptor Tyrosine Kinases as functional G Protein-Coupled Receptors. Int. Rev. Cell Mol. Biol..

[11] Girnita L., Worrall C., Takahashi S., Seregard S., Girnita A. (2014). Something old, something new and something borrowed: Emerging paradigm of insulin-like growth factor type 1 receptor (IGF-1R) signaling regulation. Cell. Mol. Life Sci..

[12] Baserga R. (1994). Oncogenes and the strategy of growth factors. Cell.

[13] Baserga R. (2005). The insulin-like growth factor-I receptor as a target for cancer therapy. Expert Opin. Ther. Targets.

[14] Achlaug L., Sarfstein R., Nagaraj K., Lapkina-Gendler L., Bruchim I., Dixit M., Laron Z., Yakar S., Werner H. (2019). Identification of ZYG11A as a candidate IGF1-dependent proto-oncogene in endometrial cancer. Oncotarget.

[15] Ulfarsson E., Karstrom A., Yin S., Girnita A., Vasilcanu D., Thoren M., Kratz G., Hillman J., Axelson M., Larsson O. (2005). Expression and growth dependency of the insulin-like growth factor I receptor in craniopharyngioma cells: A novel therapeutic approach. Clin. Cancer Res..

[16] Christopoulos P.F., Corthay A., Koutsilieris M. (2018). Aiming for the Insulin-like Growth Factor-1 system in breast cancer therapeutics. Cancer Treat. Rev..

[17] Crudden C., Girnita A., Girnita L. (2015). Targeting the IGF-1R: The Tale of the Tortoise and the Hare. Front. Endocrinol..

[18] Osher E., Macaulay V.M. (2019). Therapeutic Targeting of the IGF Axis. Cells.

[19] Gualberto A., Pollak M. (2009). Emerging role of insulin-like growth factor receptor inhibitors in oncology: Early clinical trial results and future directions. Oncogene.

[20] Burtrum D., Zhu Z., Lu D., Anderson D.M., Prewett M., Pereira D.S., Bassi R., Abdullah R., Hooper A.T., Koo H. (2003). A fully human monoclonal antibody to the insulin-like growth factor I receptor blocks ligand-dependent signaling and inhibits human tumor growth in vivo. Cancer Res..

[21] Gong Y., Yao E., Shen R., Goel A., Arcila M., Teruya-Feldstein J., Zakowski M.F., Frankel S., Peifer M., Thomas R.K. (2009). High expression levels of total IGF-1R and sensitivity of NSCLC cells in vitro to an anti-IGF-1R antibody (R1507). PLoS ONE.

[22] Baserga R. (2013). The decline and fall of the IGF-I receptor. J. Cell Physiol..

[23] Crudden C., Ilic M., Suleymanova N., Worrall C., Girnita A., Girnita L. (2015). The dichotomy of the Insulin-like growth factor 1 receptor: RTK and GPCR: Friend or foe for cancer treatment?. Growth Horm. IGF Res..

[24] Sachdev D., Li S.L., Hartell J.S., Fujita-Yamaguchi Y., Miller J.S., Yee D. (2003). A chimeric humanized single-chain antibody against the type I insulin-like growth factor (IGF) receptor renders breast cancer cells refractory to the mitogenic effects of IGF-I. Cancer Res..

[25] Girnita L., Wang M., Xie Y., Nilsson G., Dricu A., Wejde J., Larsson O. (2000). Inhibition of N-linked glycosylation down-regulates insulin-like growth factor-1 receptor at the cell surface and kills Ewing’s sarcoma cells: Therapeutic implications. Anti-Cancer Drug Des..

[26] Lu D., Zhang H., Koo H., Tonra J., Balderes P., Prewett M., Corcoran E., Mangalampalli V., Bassi R., Anselma D. (2005). A fully human recombinant IgG-like bispecific antibody to both the epidermal growth factor receptor and the insulin-like growth factor receptor for enhanced antitumor activity. J. Biol. Chem..

[27] Smith T.J. (2018). Is there potential for the approval of monoclonal antibodies to treat thyroid-associated ophthalmopathy?. Expert Opin. Orphan Drugs.

[28] Goetsch L., Gonzalez A., Leger O., Beck A., Pauwels P.J., Haeuw J.F., Corvaia N. (2005). A recombinant humanized anti-insulin-like growth factor receptor type I antibody (h7C10) enhances the antitumor activity of vinorelbine and anti-epidermal growth factor receptor therapy against human cancer xenografts. Int. J. Cancer.

[29] Wan X., Harkavy B., Shen N., Grohar P., Helman L.J. (2007). Rapamycin induces feedback activation of Akt signaling through an IGF-1R-dependent mechanism. Oncogene.

[30] Pandini G., Wurch T., Akla B., Corvaia N., Belfiore A., Goetsch L. (2007). Functional responses and in vivo anti-tumour activity of h7C10: A humanised monoclonal antibody with neutralising activity against the insulin-like growth factor-1 (IGF-1) receptor and insulin/IGF-1 hybrid receptors. Eur. J. Cancer.

[31] Cao L., Yu Y., Darko I., Currier D., Mayeenuddin L.H., Wan X., Khanna C., Helman L.J. (2008). Addiction to elevated insulin-like growth factor I receptor and initial modulation of the AKT pathway define the responsiveness of rhabdomyosarcoma to the targeting antibody. Cancer Res..

[32] Beltran P.J., Mitchell P., Chung Y.A., Cajulis E., Lu J., Belmontes B., Ho J., Tsai M.M., Zhu M., Vonderfecht S. (2009). AMG 479, a fully human anti-insulin-like growth factor receptor type I monoclonal antibody, inhibits the growth and survival of pancreatic carcinoma cells. Mol. Cancer Ther..

[33] Konijeti R., Koyama S., Gray A., Barnard R.J., Said J.W., Castor B., Elashoff D., Wan J., Beltran P.J., Calzone F.J. (2012). Effect of a low-fat diet combined with IGF-1 receptor blockade on 22Rv1 prostate cancer xenografts. Mol. Cancer Ther..

[34] Wang Y., Hailey J., Williams D., Wang Y., Lipari P., Malkowski M., Wang X., Xie L., Li G., Saha D. (2005). Inhibition of insulin-like growth factor-I receptor (IGF-IR) signaling and tumor cell growth by a fully human neutralizing anti-IGF-IR antibody. Mol. Cancer Ther..

[35] Bid H.K., Zhan J., Phelps D.A., Kurmasheva R.T., Houghton P.J. (2012). Potent inhibition of angiogenesis by the IGF-1 receptor-targeting antibody SCH717454 is reversed by IGF-2. Mol. Cancer Ther..

[36] Maloney E.K., McLaughlin J.L., Dagdigian N.E., Garrett L.M., Connors K.M., Zhou X.M., Blattler W.A., Chittenden T., Singh R. (2003). An anti-insulin-like growth factor I receptor antibody that is a potent inhibitor of cancer cell proliferation. Cancer Res..

[37] Spiliotaki M., Markomanolaki H., Mela M., Mavroudis D., Georgoulias V., Agelaki S. (2011). Targeting the insulin-like growth factor I receptor inhibits proliferation and VEGF production of non-small cell lung cancer cells and enhances paclitaxel-mediated anti-tumor effect. Lung Cancer.

[38] Cohen B.D., Baker D.A., Soderstrom C., Tkalcevic G., Rossi A.M., Miller P.E., Tengowski M.W., Wang F., Gualberto A., Beebe J.S. (2005). Combination therapy enhances the inhibition of tumor growth with the fully human anti-type 1 insulin-like growth factor receptor monoclonal antibody CP-751,871. Clin. Cancer Res..

[39] Zheng H., Shen H., Oprea I., Worrall C., Stefanescu R., Girnita A., Girnita L. (2012). beta-Arrestin-biased agonism as the central mechanism of action for insulin-like growth factor 1 receptor-targeting antibodies in Ewing’s sarcoma. Proc. Natl. Acad. Sci. USA.

[40] Kim J.G., Kang M.J., Yoon Y.K., Kim H.P., Park J., Song S.H., Han S.W., Park J.W., Kang G.H., Kang K.W. (2012). Heterodimerization of glycosylated insulin-like growth factor-1 receptors and insulin receptors in cancer cells sensitive to anti-IGF1R antibody. PLoS ONE.

[41] Leventhal P.S., Shelden E.A., Kim B., Feldman E.L. (1997). Tyrosine phosphorylation of paxillin and focal adhesion kinase during insulin-like growth factor-I-stimulated lamellipodial advance. J. Biol. Chem..

[42] Vincent E.E., Elder D.J., Curwen J., Kilgour E., Hers I., Tavare J.M. (2013). Targeting non-small cell lung cancer cells by dual inhibition of the insulin receptor and the insulin-like growth factor-1 receptor. PLoS ONE.

[43] Nguyen T.T., Sheppard A.M., Kaye P.L., Noakes P.G. (2007). IGF-I and insulin activate mitogen-activated protein kinase via the type 1 IGF receptor in mouse embryonic stem cells. Reproduction.

[44] Wang Q., Zhang F., Hong Y. (2018). Blocking of autocrine IGF-1 reduces viability of human umbilical cord mesenchymal stem cells via inhibition of the Akt/Gsk-3beta signaling pathway. Mol. Med. Rep..

[45] Wittman M., Carboni J., Attar R., Balasubramanian B., Balimane P., Brassil P., Beaulieu F., Chang C., Clarke W., Dell J. (2005). Discovery of a (1H-benzoimidazol-2-yl)-1H-pyridin-2-one (BMS-536924) inhibitor of insulin-like growth factor I receptor kinase with in vivo antitumor activity. J. Med. Chem..

[46] McKinley E.T., Bugaj J.E., Zhao P., Guleryuz S., Mantis C., Gokhale P.C., Wild R., Manning H.C. (2011). 18FDG-PET predicts pharmacodynamic response to OSI-906, a dual IGF-1R/IR inhibitor, in preclinical mouse models of lung cancer. Clin. Cancer Res..

[47] Girnita A., Girnita L., del Prete F., Bartolazzi A., Larsson O., Axelson M. (2004). Cyclolignans as inhibitors of the insulin-like growth factor-1 receptor and malignant cell growth. Cancer Res..

[48] Vasilcanu D., Girnita A., Girnita L., Vasilcanu R., Axelson M., Larsson O. (2004). The cyclolignan PPP induces activation loop-specific inhibition of tyrosine phosphorylation of the insulin-like growth factor-1 receptor. Link to the phosphatidyl inositol-3 kinase/Akt apoptotic pathway. Oncogene.

[49] Vasilcanu R., Vasilcanu D., Sehat B., Yin S., Girnita A., Axelson M., Girnita L. (2008). Insulin-like growth factor type-I receptor-dependent phosphorylation of extracellular signal-regulated kinase 1/2 but not Akt (protein kinase B) can be induced by picropodophyllin. Mol. Pharmacol..

[50] Vasilcanu R., Vasilcanu D., Rosengren L., Natalishvili N., Sehat B., Yin S., Girnita A., Axelson M., Girnita L., Larsson O. (2008). Picropodophyllin induces downregulation of the insulin-like growth factor 1 receptor: Potential mechanistic involvement of Mdm2 and beta-arrestin1. Oncogene.

[51] Carboni J.M., Wittman M., Yang Z., Lee F., Greer A., Hurlburt W., Hillerman S., Cao C., Cantor G.H., Dell-John J. (2009). BMS-754807, a small molecule inhibitor of insulin-like growth factor-1R/IR. Mol. Cancer Ther..

[52] Hou X., Huang F., Macedo L.F., Harrington S.C., Reeves K.A., Greer A., Finckenstein F.G., Brodie A., Gottardis M.M., Carboni J.M. (2011). Dual IGF-1R/InsR inhibitor BMS-754807 synergizes with hormonal agents in treatment of estrogen-dependent breast cancer. Cancer Res..

[53] Parrizas M., Gazit A., Levitzki A., Wertheimer E., LeRoith D. (1997). Specific inhibition of insulin-like growth factor-1 and insulin receptor tyrosine kinase activity and biological function by tyrphostins. Endocrinology.

[54] Wen B., Deutsch E., Marangoni E., Frascona V., Maggiorella L., Abdulkarim B., Chavaudra N., Bourhis J. (2001). Tyrphostin AG 1024 modulates radiosensitivity in human breast cancer cells. Br. J. Cancer.

[55] Von Willebrand M., Zacksenhaus E., Cheng E., Glazer P., Halaban R. (2003). The tyrphostin AG1024 accelerates the degradation of phosphorylated forms of retinoblastoma protein (pRb) and restores pRb tumor suppressive function in melanoma cells. Cancer Res..

[56] Garcia-Echeverria C., Pearson M.A., Marti A., Meyer T., Mestan J., Zimmermann J., Gao J., Brueggen J., Capraro H.G., Cozens R. (2004). In vivo antitumor activity of NVP-AEW541-A novel, potent, and selective inhibitor of the IGF-IR kinase. Cancer Cell.

[57] Tsushima H., Morimoto S., Fujishiro M., Yoshida Y., Hayakawa K., Hirai T., Miyashita T., Ikeda K., Yamaji K., Takamori K. (2017). Kinase inhibitors of the IGF-1R as a potential therapeutic agent for rheumatoid arthritis. Autoimmunity.

[58] Scotlandi K., Manara M.C., Nicoletti G., Lollini P.L., Lukas S., Benini S., Croci S., Perdichizzi S., Zambelli D., Serra M. (2005). Antitumor activity of the insulin-like growth factor-I receptor kinase inhibitor NVP-AEW541 in musculoskeletal tumors. Cancer Res..

[59] Schwartz G.K., Dickson M.A., LoRusso P.M., Sausville E.A., Maekawa Y., Watanabe Y., Kashima N., Nakashima D., Akinaga S. (2016). Preclinical and first-in-human phase I studies of KW-2450, an oral tyrosine kinase inhibitor with insulin-like growth factor receptor-1/insulin receptor selectivity. Cancer Sci..

[60] Ponka P., Lok C.N. (1999). The transferrin receptor: Role in health and disease. Int. J. Biochem. Cell Biol..

[61] Robinson M.S. (1989). Cloning of cDNAs encoding two related 100-kD coated vesicle proteins (alpha-adaptins). J. Cell Biol..

[62] Koenig J.A., Edwardson J.M. (1997). Endocytosis and recycling of G protein-coupled receptors. Trends Pharm. Sci..

[63] Goh L.K., Sorkin A. (2013). Endocytosis of receptor tyrosine kinases. Cold Spring Harb. Perspect. Biol..

[64] Lee P.S., Wang Y., Dominguez M.G., Yeung Y.G., Murphy M.A., Bowtell D.D., Stanley E.R. (1999). The Cbl protooncoprotein stimulates CSF-1 receptor multiubiquitination and endocytosis, and attenuates macrophage proliferation. EMBO J..

[65] Stoscheck C.M., Carpenter G. (1984). Characterization of the metabolic turnover of epidermal growth factor receptor protein in A-431 cells. J. Cell Physiol..

[66] Siegfried E., Korman N., Molina C., Kianifard F., Abrams K. (2006). Safety and efficacy of early intervention with pimecrolimus cream 1% combined with corticosteroids for major flares in infants and children with atopic dermatitis. J. Dermatol. Treat..

[67] Reed B.C., Lane M.D. (1980). Insulin receptor synthesis and turnover in differentiating 3T3-L1 preadipocytes. Proc. Natl. Acad. Sci. USA.

[68] Girnita L., Takahashi S.I., Crudden C., Fukushima T., Worrall C., Furuta H., Yoshihara H., Hakuno F., Girnita A. (2016). Chapter Seven - When Phosphorylation Encounters Ubiquitination: A Balanced Perspective on IGF-1R Signaling. Prog. Mol. Biol. Transl. Sci..

[69] Sehat B., Andersson S., Vasilcanu R., Girnita L., Larsson O. (2007). Role of ubiquitination in IGF-1 receptor signaling and degradation. PLoS ONE.

[70] Gasbarri A., Del Prete F., Girnita L., Martegani M.P., Natali P.G., Bartolazzi A. (2003). CD44s adhesive function spontaneous and PMA-inducible CD44 cleavage are regulated at post-translational level in cells of melanocytic lineage. Melanoma Res..

[71] Pickart C.M. (2001). Mechanisms underlying ubiquitination. Annu. Rev. Biochem..

[72] Weissman A.M. (2001). Themes and variations on ubiquitylation. Nat. Rev. Mol. Cell Biol..

[73] Kulathu Y., Komander D. (2012). Atypical ubiquitylation—The unexplored world of polyubiquitin beyond Lys48 and Lys63 linkages. Nat. Rev. Mol. Cell Biol..

[74] Trempe J.F. (2011). Reading the ubiquitin postal code. Curr. Opin. Struct. Biol..

[75] Mao Y., Shang Y., Pham V.C., Ernst J.A., Lill J.R., Scales S.J., Zha J. (2011). Polyubiquitination of insulin-like growth factor I receptor (IGF-IR) activation loop promotes antibody-induced receptor internalization and down-regulation. J. Biol. Chem..

[76] Girnita L., Girnita A., Larsson O. (2003). Mdm2-dependent ubiquitination and degradation of the insulin-like growth factor 1 receptor. Proc. Natl. Acad. Sci. USA.

[77] Sepp-Lorenzino L., Ma Z., Lebwohl D.E., Vinitsky A., Rosen N. (1995). Herbimycin A induces the 20 S proteasome- and ubiquitin-dependent degradation of receptor tyrosine kinases. J. Biol. Chem..

[78] Zhang Y., Goodfellow R., Li Y., Yang S., Winters C.J., Thiel K.W., Leslie K.K., Yang B. (2015). NEDD4 ubiquitin ligase is a putative oncogene in endometrial cancer that activates IGF-1R/PI3K/Akt signaling. Gynecol. Oncol..

[79] Sehat B., Andersson S., Girnita L., Larsson O. (2008). Identification of c-Cbl as a new ligase for insulin-like growth factor-I receptor with distinct roles from Mdm2 in receptor ubiquitination and endocytosis. Cancer Res..

[80] Xu Y.M., Wang H.J., Chen F., Guo W.H., Wang Y.Y., Li H.Y., Tang J.H., Ding Y., Shen Y.C., Li M. (2015). HRD1 suppresses the growth and metastasis of breast cancer cells by promoting IGF-1R degradation. Oncotarget.

[81] Girnita L., Shenoy S.K., Sehat B., Vasilcanu R., Vasilcanu D., Girnita A., Lefkowitz R.J., Larsson O. (2007). Beta-arrestin and Mdm2 mediate IGF-1 receptor-stimulated ERK activation and cell cycle progression. J. Biol. Chem..

[82] Monami G., Emiliozzi V., Morrione A. (2008). Grb10/Nedd4-mediated multiubiquitination of the insulin-like growth factor receptor regulates receptor internalization. J. Cell Physiol..

[83] Larsson O., Girnita A., Girnita L. (2005). Role of insulin-like growth factor 1 receptor signalling in cancer. Br. J. Cancer.

[84] Gorden P., Carpentier J.L., Cohen S., Orci L. (1978). Epidermal growth factor: Morphological demonstration of binding, internalization, and lysosomal association in human fibroblasts. Proc. Natl. Acad. Sci. USA.

[85] Beattie E.C., Howe C.L., Wilde A., Brodsky F.M., Mobley W.C. (2000). NGF signals through TrkA to increase clathrin at the plasma membrane and enhance clathrin-mediated membrane trafficking. J. Neurosci..

[86] Bogdanovic E., Coombs N., Dumont D.J. (2009). Oligomerized Tie2 localizes to clathrin-coated pits in response to angiopoietin-1. Histochem. Cell Biol..

[87] Haigler H.T., McKanna J.A., Cohen S. (1979). Rapid stimulation of pinocytosis in human carcinoma cells A-431 by epidermal growth factor. J. Cell Biol..

[88] Beguinot L., Lyall R.M., Willingham M.C., Pastan I. (1984). Down-regulation of the epidermal growth factor receptor in KB cells is due to receptor internalization and subsequent degradation in lysosomes. Proc. Natl. Acad. Sci. USA.

[89] Miller K., Beardmore J., Kanety H., Schlessinger J., Hopkins C.R. (1986). Localization of the epidermal growth factor (EGF) receptor within the endosome of EGF-stimulated epidermoid carcinoma (A431) cells. J. Cell Biol..

[90] Hopkins C.R., Gibson A., Shipman M., Miller K. (1990). Movement of internalized ligand-receptor complexes along a continuous endosomal reticulum. Nature.

[91] Wiley H.S. (1988). Anomalous binding of epidermal growth factor to A431 cells is due to the effect of high receptor densities and a saturable endocytic system. J. Cell Biol..

[92] Sigismund S., Woelk T., Puri C., Maspero E., Tacchetti C., Transidico P., Di Fiore P.P., Polo S. (2005). Clathrin-independent endocytosis of ubiquitinated cargos. Proc. Natl. Acad. Sci. USA.

[93] Backer J.M., Shoelson S.E., Haring E., White M.F. (1991). Insulin receptors internalize by a rapid, saturable pathway requiring receptor autophosphorylation and an intact juxtamembrane region. J. Cell Biol..

[94] Prager D., Li H.L., Yamasaki H., Melmed S. (1994). Human insulin-like growth factor I receptor internalization. Role of the juxtamembrane domain. J. Biol. Chem..

[95] Belfiore A., Malaguarnera R., Nicolosi M.L., Lappano R., Ragusa M., Morrione A., Vella V. (2018). A novel functional crosstalk between DDR1 and the IGF axis and its relevance for breast cancer. Cell Adhes. Migr..

[96] Stanicka J., Rieger L., O’Shea S., Cox O., Coleman M., O’Flanagan C., Addario B., McCabe N., Kennedy R., O’Connor R. (2018). FES-related tyrosine kinase activates the insulin-like growth factor-1 receptor at sites of cell adhesion. Oncogene.

[97] Salani B., Passalacqua M., Maffioli S., Briatore L., Hamoudane M., Contini P., Cordera R., Maggi D. (2010). IGF-IR internalizes with Caveolin-1 and PTRF/Cavin in HaCat cells. PLoS ONE.

[98] Suleymanova N., Crudden C., Shibano T., Worrall C., Oprea I., Tica A., Calin G.A., Girnita A., Girnita L. (2017). Functional antagonism of beta-arrestin isoforms balance IGF-1R expression and signalling with distinct cancer-related biological outcomes. Oncogene.

[99] Worrall C., Suleymanova N., Crudden C., Trocoli Drakensjo I., Candrea E., Nedelcu D., Takahashi S.I., Girnita L., Girnita A. (2017). Unbalancing p53/Mdm2/IGF-1R axis by Mdm2 activation restrains the IGF-1-dependent invasive phenotype of skin melanoma. Oncogene.

[100] Yoshihara H., Fukushima T., Hakuno F., Saeki Y., Tanaka K., Ito A., Yoshida M., Iemura S., Natsume T., Asano T. (2012). Insulin/insulin-like growth factor (IGF) stimulation abrogates an association between a deubiquitinating enzyme USP7 and insulin receptor substrates (IRSs) followed by proteasomal degradation of IRSs. Biochem. Biophys. Res. Commun..

[101] Wollert T., Hurley J.H. (2010). Molecular mechanism of multivesicular body biogenesis by ESCRT complexes. Nature.

[102] Sorkin A., Krolenko S., Kudrjavtceva N., Lazebnik J., Teslenko L., Soderquist A.M., Nikolsky N. (1991). Recycling of epidermal growth factor-receptor complexes in A431 cells: Identification of dual pathways. J. Cell Biol..

[103] Grant B.D., Donaldson J.G. (2009). Pathways and mechanisms of endocytic recycling. Nat. Rev. Mol. Cell Biol..

[104] Huecksteadt T., Olefsky J.M., Brandenberg D., Heidenreich K.A. (1986). Recycling of photoaffinity-labeled insulin receptors in rat adipocytes. Dissociation of insulin-receptor complexes is not required for receptor recycling. J. Biol. Chem..

[105] Pan B.T., Johnstone R.M. (1983). Fate of the transferrin receptor during maturation of sheep reticulocytes in vitro: Selective externalization of the receptor. Cell.

[106] Raulf N., Lucarelli P., Thavaraj S., Brown S., Vicencio J.M., Sauter T., Tavassoli M. (2018). Annexin A1 regulates EGFR activity and alters EGFR-containing tumour-derived exosomes in head and neck cancers. Eur. J. Cancer.

[107] Figueroa J.M., Skog J., Akers J., Li H., Komotar R., Jensen R., Ringel F., Yang I., Kalkanis S., Thompson R. (2017). Detection of wild-type EGFR amplification and EGFRvIII mutation in CSF-derived extracellular vesicles of glioblastoma patients. Neuro-oncology.

[108] Kharmate G., Hosseini-Beheshti E., Caradec J., Chin M.Y., Tomlinson Guns E.S. (2016). Epidermal Growth Factor Receptor in Prostate Cancer Derived Exosomes. PLoS ONE.

[109] Adamczyk K.A., Klein-Scory S., Tehrani M.M., Warnken U., Schmiegel W., Schnolzer M., Schwarte-Waldhoff I. (2011). Characterization of soluble and exosomal forms of the EGFR released from pancreatic cancer cells. Life Sci..

[110] DeRita R.M., Zerlanko B., Singh A., Lu H., Iozzo R.V., Benovic J.L., Languino L.R. (2017). c-Src, Insulin-Like Growth Factor I Receptor, G-Protein-Coupled Receptor Kinases and Focal Adhesion Kinase are Enriched Into Prostate Cancer Cell Exosomes. J. Cell. Biochem..

[111] Tomasoni S., Longaretti L., Rota C., Morigi M., Conti S., Gotti E., Capelli C., Introna M., Remuzzi G., Benigni A. (2013). Transfer of growth factor receptor mRNA via exosomes unravels the regenerative effect of mesenchymal stem cells. Stem Cells Dev..

[112] Peinado H., Aleckovic M., Lavotshkin S., Matei I., Costa-Silva B., Moreno-Bueno G., Hergueta-Redondo M., Williams C., Garcia-Santos G., Ghajar C. (2012). Melanoma exosomes educate bone marrow progenitor cells toward a pro-metastatic phenotype through MET. Nat. Med..

[113] Pyne N.J., Pyne S. (2011). Receptor tyrosine kinase-G-protein-coupled receptor signalling platforms: Out of the shadow?. Trends Pharm. Sci..

[114] Hupfeld C.J., Olefsky J.M. (2007). Regulation of receptor tyrosine kinase signaling by GRKs and beta-arrestins. Annu. Rev. Physiol..

[115] Dalle S., Imamura T., Rose D.W., Worrall D.S., Ugi S., Hupfeld C.J., Olefsky J.M. (2002). Insulin induces heterologous desensitization of G-protein-coupled receptor and insulin-like growth factor I signaling by downregulating beta-arrestin-1. Mol. Cell Biol..

[116] Hauser A.S., Attwood M.M., Rask-Andersen M., Schioth H.B., Gloriam D.E. (2017). Trends in GPCR drug discovery: New agents, targets and indications. Nat. Rev. Drug Discov..

[117] Pierce K.L., Premont R.T., Lefkowitz R.J. (2002). Seven-transmembrane receptors. Nat. Rev. Mol. Cell Biol..

[118] Chan H.C.S., Li Y., Dahoun T., Vogel H., Yuan S. (2019). New Binding Sites, New Opportunities for GPCR Drug Discovery. Trends Biochem. Sci..

[119] Wise A., Gearing K., Rees S. (2002). Target validation of G-protein coupled receptors. Drug Discov. Today.

[120] Drews J. (2000). Drug discovery: A historical perspective. Science.

[121] Wu P., Nielsen T.E., Clausen M.H. (2016). Small-molecule kinase inhibitors: An analysis of FDA-approved drugs. Drug Discov. Today.

[122] Pavlos N.J., Friedman P.A. (2017). GPCR Signaling and Trafficking: The Long and Short of It. Trends Endocrinol. Metab..

[123] Reiter E., Lefkowitz R.J. (2006). GRKs and beta-arrestins: Roles in receptor silencing, trafficking and signaling. Trends Endocrinol. Metab..

[124] Zheng H., Worrall C., Shen H., Issad T., Seregard S., Girnita A., Girnita L. (2012). Selective recruitment of G protein-coupled receptor kinases (GRKs) controls signaling of the insulin-like growth factor 1 receptor. Proc. Natl. Acad. Sci. USA.

[125] Nobles K.N., Xiao K., Ahn S., Shukla A.K., Lam C.M., Rajagopal S., Strachan R.T., Huang T.Y., Bressler E.A., Hara M.R. (2011). Distinct phosphorylation sites on the beta(2)-adrenergic receptor establish a barcode that encodes differential functions of beta-arrestin. Sci. Signal..

[126] Cahill T.J., Thomsen A.R., Tarrasch J.T., Plouffe B., Nguyen A.H., Yang F., Huang L.Y., Kahsai A.W., Bassoni D.L., Gavino B.J. (2017). Distinct conformations of GPCR-beta-arrestin complexes mediate desensitization, signaling, and endocytosis. Proc. Natl. Acad. Sci. USA.

[127] Kahsai A.W., Pani B., Lefkowitz R.J. (2018). GPCR signaling: Conformational activation of arrestins. Cell Res..

[128] Oldham W.M., Hamm H.E. (2008). Heterotrimeric G protein activation by G-protein-coupled receptors. Nat. Rev. Mol. Cell Biol..

[129] Lefkowitz R.J. (2004). Historical review: A brief history and personal retrospective of seven-transmembrane receptors. Trends Pharm. Sci..

[130] Luttrell L.M., Wang J., Plouffe B., Smith J.S., Yamani L., Kaur S., Jean-Charles P.Y., Gauthier C., Lee M.H., Pani B. (2018). Manifold roles of beta-arrestins in GPCR signaling elucidated with siRNA and CRISPR/Cas9. Sci. Signal..

[131] Pitcher J.A., Freedman N.J., Lefkowitz R.J. (1998). G protein-coupled receptor kinases. Annu. Rev. Biochem..

[132] Kuhn H., Dreyer W.J. (1972). Light dependent phosphorylation of rhodopsin by ATP. FEBS Lett..

[133] Bownds D., Dawes J., Miller J., Stahlman M. (1972). Phosphorylation of frog photoreceptor membranes induced by light. Nat. New Biol..

[134] Weller M., Virmaux N., Mandel P. (1975). Light-stimulated phosphorylation of rhodopsin in the retina: The presence of a protein kinase that is specific for photobleached rhodopsin. Proc. Natl. Acad. Sci. USA.

[135] Benovic J.L., Strasser R.H., Caron M.G., Lefkowitz R.J. (1986). Beta-adrenergic receptor kinase: Identification of a novel protein kinase that phosphorylates the agonist-occupied form of the receptor. Proc. Natl. Acad. Sci. USA.

[136] Watari K., Nakaya M., Kurose H. (2014). Multiple functions of G protein-coupled receptor kinases. J. Mol. Signal..

[137] Nogues L., Palacios-Garcia J., Reglero C., Rivas V., Neves M., Ribas C., Penela P., Mayor F. (2018). G protein-coupled receptor kinases (GRKs) in tumorigenesis and cancer progression: GPCR regulators and signaling hubs. Semin. Cancer Biol..

[138] Leroux A.E., Schulze J.O., Biondi R.M. (2018). AGC kinases, mechanisms of regulation and innovative drug development. Semin. Cancer Biol..

[139] Gurevich V.V., Gurevich E.V. (2019). GPCR Signaling Regulation: The Role of GRKs and Arrestins. Front. Pharm..

[140] Mushegian A., Gurevich V.V., Gurevich E.V. (2012). The origin and evolution of G protein-coupled receptor kinases. PLoS ONE.

[141] Rajagopal S., Shenoy S.K. (2018). GPCR desensitization: Acute and prolonged phases. Cell Signal..

[142] Premont R.T., Inglese J., Lefkowitz R.J. (1995). Protein kinases that phosphorylate activated G protein-coupled receptors. FASEB J..

[143] Steury M.D., McCabe L.R., Parameswaran N. (2017). G Protein-Coupled Receptor Kinases in the Inflammatory Response and Signaling. Adv. Immunol..

[144] Gilman A.G. (1987). G proteins: Transducers of receptor-generated signals. Annu. Rev. Biochem..

[145] Touhara K., Inglese J., Pitcher J.A., Shaw G., Lefkowitz R.J. (1994). Binding of G protein beta gamma-subunits to pleckstrin homology domains. J. Biol. Chem..

[146] Premont R.T., Gainetdinov R.R. (2007). Physiological roles of G protein-coupled receptor kinases and arrestins. Annu. Rev. Physiol..

[147] Gurevich E.V., Tesmer J.J., Mushegian A., Gurevich V.V. (2012). G protein-coupled receptor kinases: More than just kinases and not only for GPCRs. Pharm. Ther..

[148] Gurevich V.V., Gurevich E.V. (2013). Structural determinants of arrestin functions. Prog. Mol. Biol. Transl. Sci..

[149] Conner D.A., Mathier M.A., Mortensen R.M., Christe M., Vatner S.F., Seidman C.E., Seidman J.G. (1997). beta-Arrestin1 knockout mice appear normal but demonstrate altered cardiac responses to beta-adrenergic stimulation. Circ. Res..

[150] Bohn L.M., Lefkowitz R.J., Gainetdinov R.R., Peppel K., Caron M.G., Lin F.T. (1999). Enhanced morphine analgesia in mice lacking beta-arrestin 2. Science.

[151] Shenoy S.K., Modi A.S., Shukla A.K., Xiao K., Berthouze M., Ahn S., Wilkinson K.D., Miller W.E., Lefkowitz R.J. (2009). Beta-arrestin-dependent signaling and trafficking of 7-transmembrane receptors is reciprocally regulated by the deubiquitinase USP33 and the E3 ligase Mdm2. Proc. Natl. Acad. Sci. USA.

[152] Shenoy S.K., Barak L.S., Xiao K., Ahn S., Berthouze M., Shukla A.K., Luttrell L.M., Lefkowitz R.J. (2007). Ubiquitination of beta-arrestin links seven-transmembrane receptor endocytosis and ERK activation. J. Biol. Chem..

[153] Lefkowitz R.J., Shenoy S.K. (2005). Transduction of receptor signals by beta-arrestins. Science.

[154] Lefkowitz R.J. (2013). Arrestins come of age: A personal historical perspective. Prog. Mol. Biol. Transl. Sci..

[155] Butcher A.J., Prihandoko R., Kong K.C., McWilliams P., Edwards J.M., Bottrill A., Mistry S., Tobin A.B. (2011). Differential G-protein-coupled receptor phosphorylation provides evidence for a signaling bar code. J. Biol. Chem..

[156] Carman C.V., Benovic J.L. (1998). G-protein-coupled receptors: Turn-ons and turn-offs. Curr. Opin. Neurobiol..

[157] Gurevich V.V., Gurevich E.V. (2006). The structural basis of arrestin-mediated regulation of G-protein-coupled receptors. Pharmacol. Ther..

[158] Gurevich V.V., Gurevich E.V. (2004). The molecular acrobatics of arrestin activation. Trends Pharm. Sci..

[159] Choi M., Staus D.P., Wingler L.M., Ahn S., Pani B., Capel W.D., Lefkowitz R.J. (2018). G protein-coupled receptor kinases (GRKs) orchestrate biased agonism at the beta2-adrenergic receptor. Sci. Signal..

[160] Wingler L.M., Elgeti M., Hilger D., Latorraca N.R., Lerch M.T., Staus D.P., Dror R.O., Kobilka B.K., Hubbell W.L., Lefkowitz R.J. (2019). Angiotensin Analogs with Divergent Bias Stabilize Distinct Receptor Conformations. Cell.

[161] Shenoy S.K. (2014). Arrestin interaction with E3 ubiquitin ligases and deubiquitinases: Functional and therapeutic implications. Handb. Exp. Pharm..

[162] Gao H., Sun Y., Wu Y., Luan B., Wang Y., Qu B., Pei G. (2004). Identification of beta-arrestin2 as a G protein-coupled receptor-stimulated regulator of NF-kappaB pathways. Mol. Cell.

[163] Shenoy S.K., Drake M.T., Nelson C.D., Houtz D.A., Xiao K., Madabushi S., Reiter E., Premont R.T., Lichtarge O., Lefkowitz R.J. (2006). beta-arrestin-dependent, G protein-independent ERK1/2 activation by the beta2 adrenergic receptor. J. Biol. Chem..

[164] Luttrell L.M., Ferguson S.S., Daaka Y., Miller W.E., Maudsley S., Della Rocca G.J., Lin F., Kawakatsu H., Owada K., Luttrell D.K. (1999). Beta-arrestin-dependent formation of beta2 adrenergic receptor-Src protein kinase complexes. Science.

[165] Ahn S., Kim J., Hara M.R., Ren X.R., Lefkowitz R.J. (2009). {beta}-Arrestin-2 Mediates Anti-apoptotic Signaling through Regulation of BAD Phosphorylation. J. Biol. Chem..

[166] Kendall R.T., Lee M.H., Pleasant D.L., Robinson K., Kuppuswamy D., McDermott P.J., Luttrell L.M. (2014). Arrestin-dependent angiotensin AT1 receptor signaling regulates Akt and mTor-mediated protein synthesis. J. Biol. Chem..

[167] Coffa S., Breitman M., Spiller B.W., Gurevich V.V. (2011). A single mutation in arrestin-2 prevents ERK1/2 activation by reducing c-Raf1 binding. Biochemistry.

[168] Shenoy S.K., Lefkowitz R.J. (2011). beta-Arrestin-mediated receptor trafficking and signal transduction. Trends Pharm. Sci.

[169] Shenoy S.K., Lefkowitz R.J. (2005). Receptor regulation: Beta-arrestin moves up a notch. Nat. Cell Biol..

[170] Shenoy S.K., Lefkowitz R.J. (2005). Seven-transmembrane receptor signaling through beta-arrestin. Sci. Stke: Signal Transduct. Knowl. Environ..

[171] Oakley R.H., Laporte S.A., Holt J.A., Caron M.G., Barak L.S. (2000). Differential affinities of visual arrestin, beta arrestin1, and beta arrestin2 for G protein-coupled receptors delineate two major classes of receptors. J. Biol. Chem..

[172] Andersson E.R. (2012). The role of endocytosis in activating and regulating signal transduction. Cell. Mol. Life Sci..

[173] Luo Y., Cheng Z., Dixon C.J., Hall J.F., Taylor E., Boarder M.R. (2011). Endosomal signalling of epidermal growth factor receptors contributes to EGF-stimulated cell cycle progression in primary hepatocytes. Eur. J. Pharmacol..

[174] Huynh J., Kwa M.Q., Cook A.D., Hamilton J.A., Scholz G.M. (2012). CSF-1 receptor signalling from endosomes mediates the sustained activation of Erk1/2 and Akt in macrophages. Cell Signal..

[175] Girnita L., Girnita A., Brodin B., Xie Y., Nilsson G., Dricu A., Lundeberg J., Wejde J., Bartolazzi A., Wiman K.G. (2000). Increased expression of insulin-like growth factor I receptor in malignant cells expressing aberrant p53: Functional impact. Cancer Res..

[176] Girnita L., Shenoy S.K., Sehat B., Vasilcanu R., Girnita A., Lefkowitz R.J., Larsson O. (2005). β-Arrestin is crucial for ubiquitination and down-regulation of the insulin-like growth factor-1 receptor by acting as adaptor for the MDM2 E3 ligase. J. Biol. Chem..

[177] Economou M.A., Wu J., Vasilcanu D., Rosengren L., All-Ericsson C., van der Ploeg I., Menu E., Girnita L., Axelson M., Larsson O. (2008). Inhibition of VEGF secretion and experimental choroidal neovascularization by picropodophyllin (PPP), an inhibitor of the insulin-like growth factor-1 receptor. Investig. Ophthalmol. Vis. Sci..

[178] Girnita A., Zheng H., Gronberg A., Girnita L., Stahle M. (2012). Identification of the cathelicidin peptide LL-37 as agonist for the type I insulin-like growth factor receptor. Oncogene.

[179] Werner H., Karnieli E., Rauscher F.J., LeRoith D. (1996). Wild-type and mutant p53 differentially regulate transcription of the insulin-like growth factor I receptor gene. Proc. Natl. Acad. Sci. USA.

[180] Ohlsson C., Kley N., Werner H., LeRoith D. (1998). p53 regulates insulin-like growth factor-I (IGF-I) receptor expression and IGF-I-induced tyrosine phosphorylation in an osteosarcoma cell line: Interaction between p53 and Sp1. Endocrinology.

[181] Werner H., Sarfstein R., LeRoith D., Bruchim I. (2016). Insulin-like Growth Factor 1 Signaling Axis Meets p53 Genome Protection Pathways. Front. Oncol..

[182] Suleymanova N., Crudden C., Worrall C., Dricu A., Girnita A., Girnita L. (2017). Enhanced response of melanoma cells to MEK inhibitors following unbiased IGF-1R down-regulation. Oncotarget.

[183] Luttrell L.M., van Biesen T., Hawes B.E., Koch W.J., Touhara K., Lefkowitz R.J. (1995). G beta gamma subunits mediate mitogen-activated protein kinase activation by the tyrosine kinase insulin-like growth factor 1 receptor. J. Biol. Chem..

[184] Vitale M., Prestat G., Lopes D., Madec D., Kammerer C., Poli G., Girnita L. (2008). New picropodophyllin analogs via palladium-catalyzed allylic alkylation-Hiyama cross-coupling sequences. J. Org. Chem..

[185] Vasilcanu D., Weng W.H., Girnita A., Lui W.O., Vasilcanu R., Axelson M., Larsson O., Larsson C., Girnita L. (2006). The insulin-like growth factor-1 receptor inhibitor PPP produces only very limited resistance in tumor cells exposed to long-term selection. Oncogene.

[186] Beckwith H., Yee D. (2015). Minireview: Were the IGF Signaling Inhibitors All Bad?. Mol. Endocrinol..

[187] Toretsky J.A., Kalebic T., Blakesley V., LeRoith D., Helman L.J. (1997). The insulin-like growth factor-I receptor is required for EWS/FLI-1 transformation of fibroblasts. J. Biol. Chem..

[188] Cironi L., Riggi N., Provero P., Wolf N., Suva M.L., Suva D., Kindler V., Stamenkovic I. (2008). IGF1 is a common target gene of Ewing’s sarcoma fusion proteins in mesenchymal progenitor cells. PLoS ONE.

[189] Prieur A., Tirode F., Cohen P., Delattre O. (2004). EWS/FLI-1 silencing and gene profiling of Ewing cells reveal downstream oncogenic pathways and a crucial role for repression of insulin-like growth factor binding protein 3. Mol. Cell Biol..

[190] Chen H.X., Sharon E. (2013). IGF-1R as an anti-cancer target--trials and tribulations. Chin. J. Cancer.

[191] Tognon C.E., Sorensen P.H. (2012). Targeting the insulin-like growth factor 1 receptor (IGF1R) signaling pathway for cancer therapy. Expert Opin. Ther. Targets.

[192] Bruchim I., Attias Z., Werner H. (2009). Targeting the IGF1 axis in cancer proliferation. Expert Opin. Ther. Targets.

[193] Belfiore A., Malaguarnera R., Vella V., Lawrence M.C., Sciacca L., Frasca F., Morrione A., Vigneri R. (2017). Insulin Receptor Isoforms in Physiology and Disease: An Updated View. Endocr. Rev..

[194] Manara M.C., Garofalo C., Ferrari S., Belfiore A., Scotlandi K. (2013). Designing novel therapies against sarcomas in the era of personalized medicine and economic crisis. Curr. Pharm. Des..

[195] Holly J.M.P., Biernacka K., Perks C.M. (2019). The Neglected Insulin: IGF-II, a Metabolic Regulator with Implications for Diabetes, Obesity, and Cancer. Cells.

[196] Mosesson Y., Mills G.B., Yarden Y. (2008). Derailed endocytosis: An emerging feature of cancer. Nat. Reviews. Cancer.

